# α-Amino-β-carboxymuconate-ε-semialdehyde decarboxylase catalyzes enol/keto tautomerization of oxaloacetate

**DOI:** 10.1016/j.jbc.2024.107878

**Published:** 2024-10-11

**Authors:** Yu Yang, Ian Davis, Ryan A. Altman, Aimin Liu

**Affiliations:** 1 State Key Laboratory of Biocatalysis and Enzyme Engineering, Hubei Collaborative Innovation Center for Green Transformation of Bio-Resources, Hubei Key Laboratory of Industrial Biotechnology, School of Life Sciences, Hubei University, Wuhan, China; 2 Department of Chemistry, University of Texas at San Antonio, San Antonio, Texas, USA; 3 Borch Department of Medicinal Chemistry and Molecular Pharmacology and Department of Chemistry, Purdue University, West Lafayette, Indiana, USA

**Keywords:** kynurenine, decarboxylase, NAD homeostasis, tryptophan metabolism, metalloenzyme

## Abstract

ACMSD (α-amino-β-carboxymuconate-ε-semialdehyde decarboxylase) is a key metalloenzyme critical for regulating *de novo* endogenous NAD^+^/NADH biosynthesis through the tryptophan-kynurenine pathway. This decarboxylase is a recognized target implicated in mitochondrial diseases and neurodegenerative disorders. However, unraveling its enzyme-substrate complex has been challenging due to its high catalytic efficiency. Here, we present a combined biochemical and structural study wherein we determined the crystal structure of ACMSD in complex with malonate. Our analysis revealed significant rearrangements in the active site, particularly in residues crucial for ACMS decarboxylation, including Arg51, Arg239∗ (a residue from an adjacent subunit), His228, and Trp194. Docking modeling studies proposed a putative ACMS binding mode. Additionally, we found that ACMSD catalyzes oxaloacetic acid (OAA) tautomerization at a rate of 6.51 ± 0.42 s^−1^ but not decarboxylation. The isomerase activity of ACMSD on OAA warrants further investigation in future biological studies. Subsequent mutagenesis studies and crystallographic analysis of the W194A variant shed light on the roles of specific second-coordination sphere residues. Our findings indicate that Arg51 and Arg239∗ are crucial for OAA tautomerization. Moreover, our comparative analysis with related isomerase superfamily members underscores a general strategy employing arginine residues to promote OAA isomerization. Given the observed isomerase activity of ACMSD on OAA and its structural similarity to ACMS, we propose that ACMSD may facilitate isomerization to ensure ACMS is in the optimal tautomeric form for subsequent decarboxylation initiated by the zinc-bound hydroxide ion. Overall, these findings deepen the understanding of the structure and function of ACMSD, offering insights into potential therapeutic interventions.

The tryptophan-kynurenine pathway plays a crucial role in the breakdown of L-tryptophan in mammalian cells and some bacteria, yielding various neurologically active molecules such as kynurenine, kynurenic acid, and quinolinic acid (QUIN, Fig. 1) (1, 2, 3, 4, 5, 6). The pathway’s unique intricacy lies in the unstable intermediate metabolites that lead to QUIN and its subsequent breakdown. This intricate process is managed by enzymes within the pathway, which demonstrate remarkable proficiency in orchestrating chemical rearrangements through tautomerizing those unstable intermediates, ensuring a smooth and controlled progression through the pathway while maintaining minor production of intended side products, such as NAD^+^/NADH (7, 8, 9). QUIN is the universal precursor for synthesizing NAD^+^/NADH, vital molecules involved in redox reactions across all living organisms (10, 11, 12, 13, 14). Although most of our NAD^+^/NADH is derived from dietary niacin, a portion is *de novo* synthesized from L-tryptophan *via* this pathway, diverging just before QUIN production. Endogenous QUIN production is beneficial in addressing NAD^+^/NADH deficiency and maintaining NAD^+^ homeostasis in liver and kidney-related mitochondrial diseases (10, 15, 16), as NAD^+^ production occurs after QUIN production. Conversely, excessive QUIN accumulation is implicated in the pathogenesis and progression of neurodegenerative diseases like Parkinson’s, Huntington’s, and Alzheimer’s (1, 2, 17, 18, 19). Therefore, precise regulation of QUIN production within the tryptophan-kynurenine pathway holds significant implications for mammalian physiology (4).

**Figure 1:**
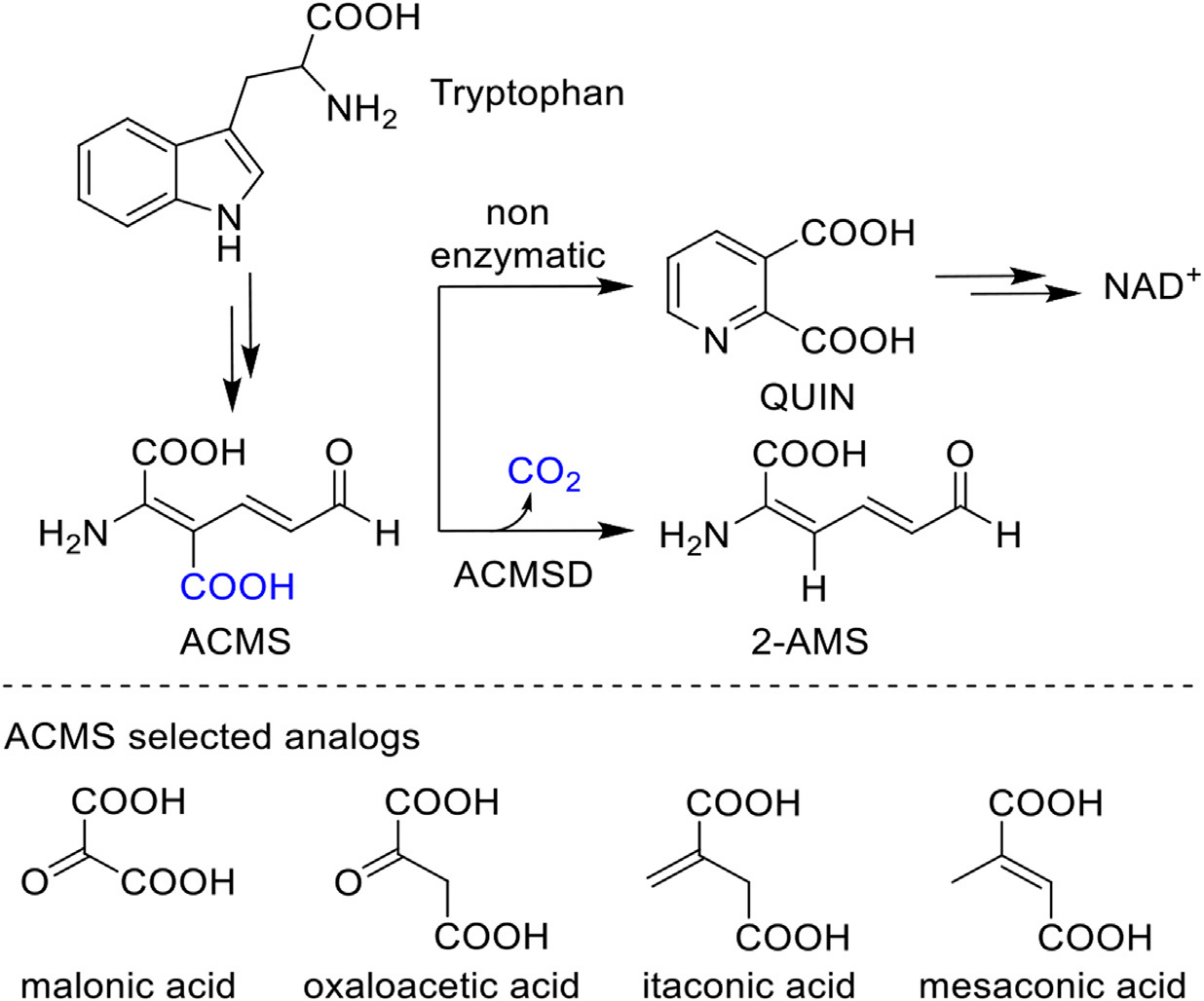
**ACMSD catalyzes the decarboxylation of ACMS.** Analogs are shown at the *bottom*.

ACMSD, the enzyme responsible for decarboxylating α-amino-β-carboxymuconate-ε-semialdehyde (ACMS) to produce α-aminomuconate-ε-semialdehyde (2-AMS, Fig. 1), plays a crucial role in controlling QUIN levels. This zinc-dependent metalloprotein is situated within the tryptophan-kynurenine pathway (20), where it competes with a spontaneous chemical reaction that converts ACMS directly to QUIN (Fig. 1). By catalyzing the removal of a carboxylate group from ACMS, ACMSD effectively channels the metabolic flow toward deep oxidation in the tricarboxylic acid (TCA) cycle (21, 22, 23). Interestingly, ACMS is highly unstable, undergoing spontaneous conversion to QUIN within minutes at room temperature and seconds at body temperature (12). Consequently, reduced ACMSD activity can lead to QUIN accumulation. Recent studies found that inhibiting ACMSD *in vivo* could redirect ACMS toward *de novo* NAD^+^ synthesis, potentially ameliorating NAD^+^ deficiency in liver and kidney-related mitochondrial diseases (10, 15, 16). This emerging role positions ACMSD as a promising target for developing drugs aimed at combating NAD^+^ deficiency-related diseases (16, 24). Understanding the mechanism of ACMSD provides valuable insights into the regulation of key intermediates within the tryptophan-kynurenine pathway, offering potential avenues for therapeutic interventions.

ACMSD is classified as a member of the amidohydrolase superfamily, representing a C–C bond-breaking activity (25, 26). Crystal structures of ACMSD from both human and bacterial sources have been elucidated (22, 27). With the cavity of ACMSD, a mononuclear Zn^2+^ ion coordinates with His9, His11, His177, Asp294, and a water molecule (residue numbering based on ACMSD from *Pseudomonas fluorescens*, *pf*ACMSD) (22). ACMSD functions as a dimer, with residue Arg239∗ from the adjacent subunit being crucial for the decarboxylation reaction (23, 28). Moreover, *pf*ACMSD employs high-order oligomers to modulate its activity further (29). Another residue in the active site, His228, plays a dual role in determining the metal selectivity and activating the metal-bound water ligand to a catalytic nucleophile (30).

The catalytic efficiency of ACMSD is remarkable, operating at a rate greater than 2.4 × 10^6^ M^−1^s^−1^ for the decarboxylation of ACMS, with product release (8.8 s^−1^) being the rate-limiting step (12). Elucidating the substrate- or product-binding modes will be instrumental in understanding the mechanism of ACMSD-catalyzed decarboxylation. Several complex structures of ACMSD have been reported. In 2009, ACMSD was found to bind the glycolic intermediate 1,3-dihydroxyacetone phosphate, suggesting a potential connection with carbohydrate metabolism (27). Additionally, a crystal structure of human ACMSD in complex with an inhibitor, pyridine-2,6-dicarboxylic acid (PDC) has also been elucidated (Fig. S1), wherein two catalytically essential Arg residues (one of which is Arg239∗ from an adjacent subunit) interact with the two catalytically carboxylic groups of the inhibitor (23). Recently, more ACMSD structures in complexes with inhibitors have been reported. A bulkier but highly potent inhibitor TES-1025 (C_18_H_13_N_3_O_3_S_2_), directly binds the Zn ion of ACMSD (16, 31), while the nonsteroid anti-inflammatory drug diflunisal binds and inhibits ACMSD (24).

In this study, we investigated the substrate binding mode and catalytic mechanism by employing substrate analogs, malonate and oxaloacetic acid (OAA). Our findings highlight the essential role of the residue Trp194 in the active site during catalysis. Furthermore, we discovered that ACMSD can catalyze the tautomerization of oxaloacetic acid (OAA), which shares a similar skeleton with ACMS. Additionally, we discuss the potential requirement for tautomerization of the substrate during ACMSD-catalyzed decarboxylation. These results provide deeper insights into the mechanism, offering valuable contributions to our understanding of the regulation of the neurologically active intermediate, QUIN, which holds significance for future studies.

## Results

### ACMSD catalyzed the tautomerization of OAA

To elucidate the decarboxylation mechanism, we explored the activity of three analogs containing the amine group substituted by a carbon atom, including itaconic acid, mesaconic acid, and OAA (Fig. 1). Surprisingly, none of these analogs exhibited catalytic decarboxylation activity when subjected to ACMSD in our activity assays (Fig. S2 and Table S1). However, as detailed below, our investigations revealed a previously unrecognized role of ACMSD in catalyzing the keto-enol tautomerization of OAA.

**Figure 2:**
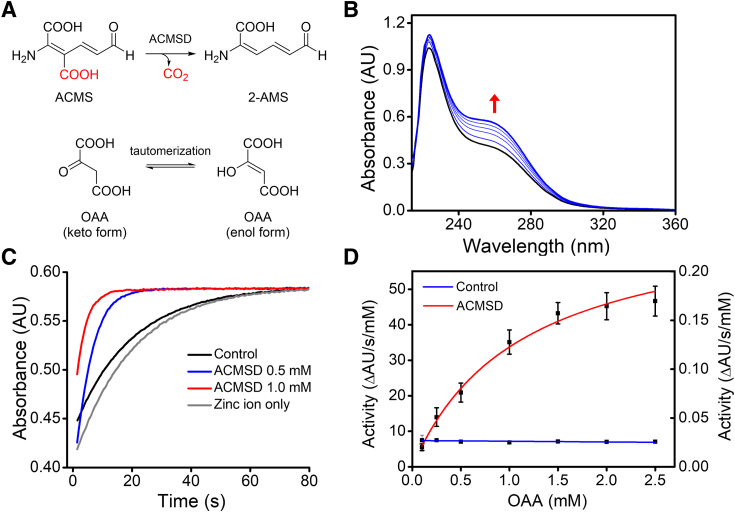
**ACMSD exhibits tautomerization activity with OAA.***A*, chemical structures of ACMS and OAA with two tautomeric forms in equilibrium. *B*, representative assay showing the ACMSD mediated the tautomerization from keto form (*blackline*) to enol form (*blueline*). *C*, ACMSD accelerates the tautomerization of OAA from keto form to its enol form compared to the control (λ = 260 nm). *D*, determination of Michaelis-Menten parameters of ACMSD towards OAA.

We found that the keto-form of OAA predominates at lower pH values and undergoes non-enzymatic equilibration to its enol form upon increasing the pH (Fig. 2*A*) (32). This transition was accompanied by an increase in absorbance between 240 and 280 nm, consistent with previous observations (Fig. 2*B*) (33). Without an enzyme (control sample), the keto form of OAA gradually converted to its enol form (Fig. 2*C*). However, the presence of ACMSD markedly accelerated the rate of keto-enol equilibrium, with the acceleration being concentration-dependent. The tautomerization rates of OAA catalyzed by ACMSD were fitted with the Michaelis-Menten equation to determine a *k*_cat_ and *K*_M_ of 6.51 ± 0.42 s^−1^ and 1120 ± 140 μM, respectively (Fig. 2*D* and Table 1). Neither free Zn^2+^ ions nor apoenzyme showed observable activity compared to the holoenzyme. These data suggested that protein-bound but not free Zn ions facilitate the tautomerization of OAA (Fig. 2*C*). Furthermore, compared to the decarboxylation of ACMS catalyzed by ACMSD, the tautomerization of OAA by ACMSD exhibited a similar *k*_cat_ value but a 49-fold increased *K*_M_ (Table 1).

**Table 1:** Kinetic parameters of ACMSD and mutants towards ACMS and OAA

Enzyme	ACMS			OAA		
	*k*_cat_ (s^−1^)	*K*_M_ (μM)	*k*_cat_/*K*_M_ (s^−1^μM^−1^)	*k*_cat_ (s^−1^)	*K*_M_ (μM)	*k*_cat_/*K*_M_ (s^−1^μM^−1^)
Wild-type	15.8 ± 1.4	22.9 ± 5.9	0.6900	6.51 ± 0.42	1120 ± 140	0.0058
W194A	0.598 ± 0.042	43.1 ± 5.7	0.0139	4.18 ± 0.25	800 ± 80	0.0052
H228A	0.111 ± 0.003	5.31 ± 0.50	0.0209	2.98 ± 0.35	570 ± 130	0.0052
H228Y	ND^a^			ND		
R51A	ND			ND		
R239A	ND			ND		

Data are presented as mean values ± standard deviation.

a ND means undetectable.

### Malonate-bound structure of ACMSD

**Figure 3:**
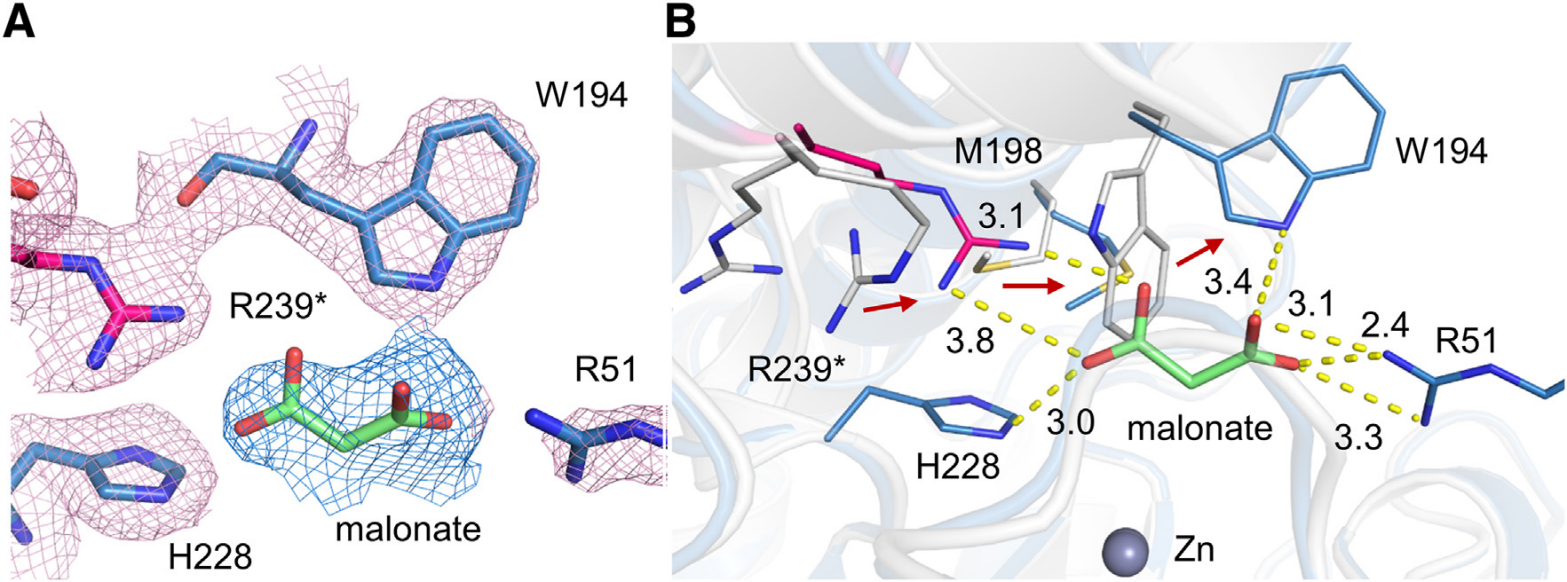
**Crystal structure of ACMSD in complex with malonate (PDB ID:****8YT1****).***A*, the omit maps (*F*_o_–*F*_c_) of malonate in *blue* and interacting residues in *pink* are contoured at 2.5 σ. Arg239∗, with its carbons shown in *pink*, is an intruding residue from the neighboring subunit. *B*, the superimposed malonate-bound ACMSD (*blue*) with the holo structure (*gray*, PDB ID: 2HBV). The key residues are shown in *sticks*, and malonate is shown in *green sticks*. The *red arrows* indicate a side chain shift upon binding malonate. The distances among atoms are shown as *yellow dashed lines* and labeled in the unit of angstrom.

We made extensive efforts to determine the structure of the binary complex of ACMS-ACMSD; however, these endeavors did not yield fruitful results. Instead, we obtained crystals when malonate was present in the refined crystallization condition and successfully solved the crystal structure of malonate-bound ACMSD at a resolution of 2.21 Å. Malonate shares similar structural features with ACMS, possessing two carboxylate groups but with one less carbon atom in between (Fig. 1). The additional electron density exhibited in the active site accommodates the malonate molecule well, establishing interactions with surrounding residues, including Arg51, Trp194, His228, and Arg239∗ (Fig. 3*A*). While the roles of Arg51, Arg239∗, and His228 in ACMS decarboxylation and the metal selectivity of ACMSD have been characterized (28, 30), the role of Trp194 remains unstudied.

Binding of malonate induced minimal structural alternations. Comparing this binary complex structure to the wild-type ACMSD structure, the RMSD value is 0.276 Å on 258 Cα in total (Fig. S3*A* and Table S2). However, residues within the active site underwent substantial conformational changes to accommodate malonate. Specifically, as depicted in Figure 3*B*, the side chain of Trp194 rotated approximately 90° to form an H-bond with the carboxylic group of malonate. Additionally, the sulfur atom of Met198 shifted by 3.9 Å upon malonate binding. To facilitate salt-bridge formation with the ligand, the two conformations of the Arg239∗ side chain in the ligand-free structure merged into a single conformation, and the previously missing electron density corresponding to the guanidinium group of Arg51 became visible (Fig. 3*A*). Notably, the conformational changes observed in Trp194 and Met198 upon malonate binding are reminiscent of those observed in diflunisal-bound *pf*ACMSD (24), and in human ACMSD bound to 1,3-dihydroxyacetone phosphate, PDC, and TES-1025 (23, 27, 31) (Fig. S1). These conformational changes in residues upon malonate binding provide valuable insights for understanding the binding mode of carboxylate-rich substrates within the active site of ACMSD, such as ACMS.

### Roles of the active site residues in ACMSD-mediated reactions

Previous studies have shown R51A, H228Y, and H239A abolish ACMS decarboxylation activity (23, 28, 30). Trp194, located approximately 5 Å away from the Zn ion, possesses a bulky side chain in the active site of the ligand-free structure, suggesting its potential participation in the ACMS decarboxylation. Here, we replaced Trp194 with alanine to examine its role in catalysis. The crystal structure of W194A was determined at the resolution of 2.02 Å (Table 2). W194A maintains the overall structure of ACMSD, with an RMSD value of 0.265 Å on 265 Cα (Fig. S3*B*). Comparative analysis revealed that mutants H228A and W194A exhibit a significant decrease in *k*_cat_ values for ACMS decarboxylation by 142-fold and 26-fold, respectively, relative to wild-type ACMSD (Table 1), indicating the essential roles of His228 and Trp194 in ACMS decarboxylation. The 1.9-fold increase in *K*_M_ value for W194A suggests a minimal role in substrate binding, potentially shielding the active site from the solvent. Given that product release is the rate-limiting step (12), Trp194 may also be involved in this process.

**Table 2:** Crystallization data collection and refinement statistics

Protein	Malonate-bound ACMSD	ACMSD W194A
PDB code	8YT1	8YT2
Data collection		
Space group	*C*222_1_	*C*222_1_
Cell dimensions a, b, c (Å)	104.3, 152.0, 153.9	104.0, 151.6, 154.1
α, β, γ (°)	90/90/90	90/90/90
Resolution	50.0–2.21	50.0–2.02
	(2.29–2.21)^a^	(2.05–2.02)
No. of observed reflections	394,895 (5969)	568,184 (3948)
Redundancy	6.6 (6.6)	7.1 (5.9)
Completeness (%)	99.0 (99.8)	99.9 (99.6)
I/sigma (I)	16.4 (2.3)	15.7 (1.6)
*R*_merge_ (%)^b^	12.4 (97.2)	12.9 (95.5)
CC_1/2_^c^	1.0 (0.95)	1.0 (0.71)
Refinement^d^		
*R*_work_	20.8	19.4
*R*_free_	25.9	23.1
RMSD bond length (Å)^e^	0.008	0.008
RMSD bond angles (°)	0.924	0.858
Ramachandran statistics^f^		
Preferred (%)	95.5	97.4
Allowed (%)	4.2	2.5
Outliers (%)	0.3	0.1
Average B-factor (Å^2^)^g^		
Protein/atoms	59.4/7749	37.9/7694
Metal/atoms	46.8/3	29.0/3
Ligand/atoms	58.0/7	-
Solvent/atoms	54.5/231	39.6/659

a Values in parentheses are for the highest resolution shell.

b $R_{\text{merge}}=\Sigma_{\text{hkl}}\Sigma i\;\left|I_{i}\left(hkl\right)-\langle I\left(hkl\right)\rangle \right|/\Sigma_{\text{hkl}}\Sigma_{i}I_{i}\left(hkl\right)$, in which the sum is over all the *i* measured reflections with equivalent miller indices hkl;⟨I(hkl)⟩ is the averaged intensity of these *i* reflections, and the grand sum is over all measured reflections in the data set.

c According to Karplus and Diederichs (52).

d All positive reflections were used in the refinement.

e According to Engh and Huber (53).

f Calculated by using MolProbity (54).

g The values were calculated using a modular *B*_average_ in CCP4.

We further investigated the impact of these active site residues on ACMSD-catalyzed tautomerization of OAA. R51A and R239A mutants did not show detectable tautomerization activity towards OAA, consistent with their roles as gatekeepers for carboxylate-rich substrates, including ACMS and OAA (Table 1). Therefore, both Arg51 and Arg239 are essential for enhancing OAA tautomerization. Interestingly, mutating His228 to Tyr abolishes the tautomerization activity, while H228A and W194A mutants exhibit comparable *k*_cat_ and *K*_M_ values to wild-type ACMSD. These findings indicate that His228 and Trp194 are not involved in catalyzing OAA tautomerization.

### An ACMS-binding mode built based on the complex structure

**Figure 4:**
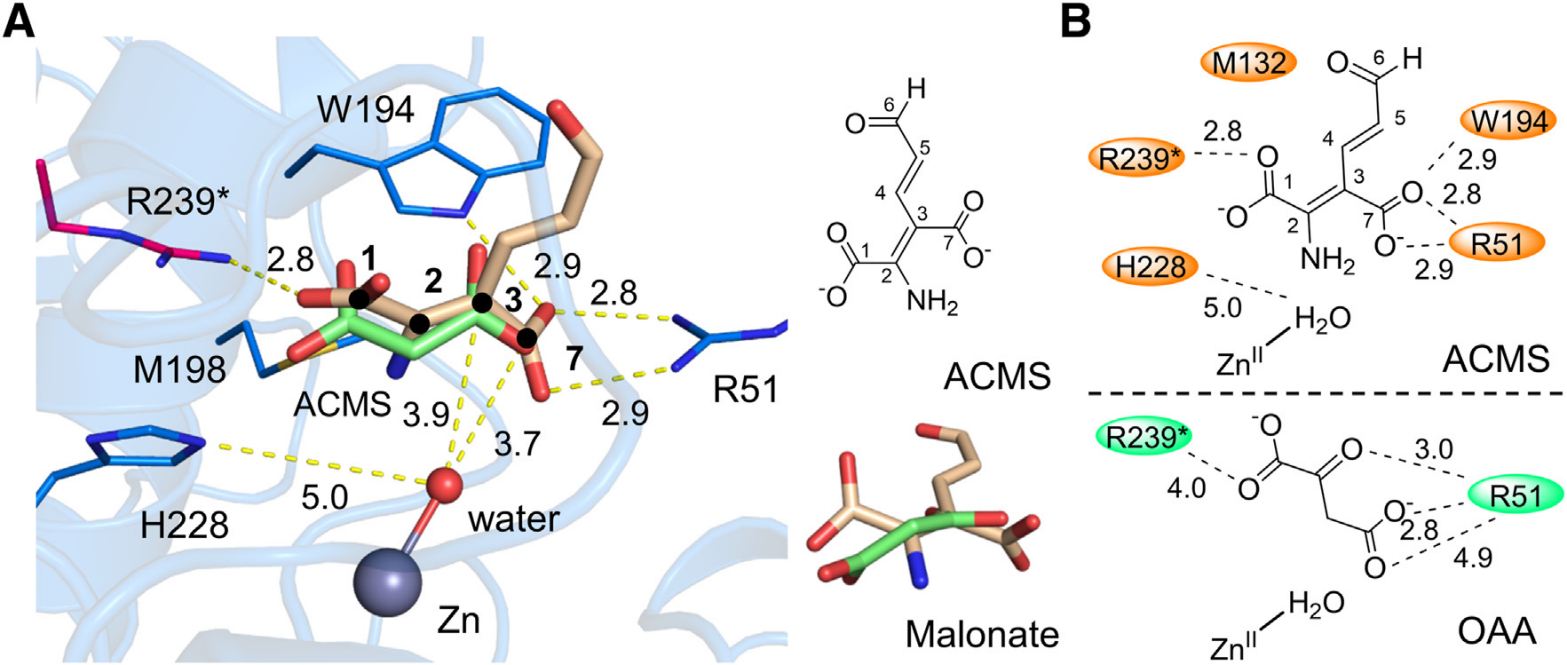
**Comparison of the malonate- and ACMS-bound ACMSD structures.***A*, structural superposition of malonate-bound crystal structure and ACMS-bound docking structure. The key residues are shown as *sticks*. The external ligands of malonate and ACMS are *green* and *orange*, respectively. The overlapped mode of malonate and ACMS in other angles is shown on the *right*. The zinc ion and solvent-derived ligands are shown as *gray* and *red balls*, respectively. The distances among atoms are shown as *yellow dashed lines* and labeled in the angstrom unit. The carbons on ACMS are labeled by bolded numbers. *B*, two-dimensional interaction diagrams for the binding modes of ACMS (*upper panel*) and OAA (*lower panel*) in ACMSD. The residues exhibiting the essential roles in the counterpart catalytic reactions are highlighted.

The lack of information on the ACMS-bound structure hampers the elucidation of the decarboxylation mechanism catalyzed by ACMSD. The substrate analog malonate-bound structure provides insights into the potential binding mode of ACMS. The docking experiment using DS2020 (details in Experimental procedures) allowed us to generate an ACMS-bound structure with the best docking score (Fig. 4*A*). In the model, the “head” moiety of ACMS, containing two carboxylic groups, is located at the bottom of the pocket and does not bind to the Zn ion, while the “tail” of ACMS (C3–C6) points towards the surface. As anticipated, the two carboxylic groups of ACMS (C1 and C7) form salt bridges with Arg51 and Arg239∗ (Table S3), while also interacting with Trp194 (Fig. 4*B*). The orientation of ACMS in the binding pocket is consistent with the binding behavior of malonate in ACMSD (Fig. 3), supporting the reliability of the docking mode. The Zn-bound water points to the leaving carboxylic group (C7). These results are consistent with the fact that, after deprotonation, the metal-bound water molecule becomes a nucleophile that attacks ACMS during decarboxylation.

**Figure 5:**
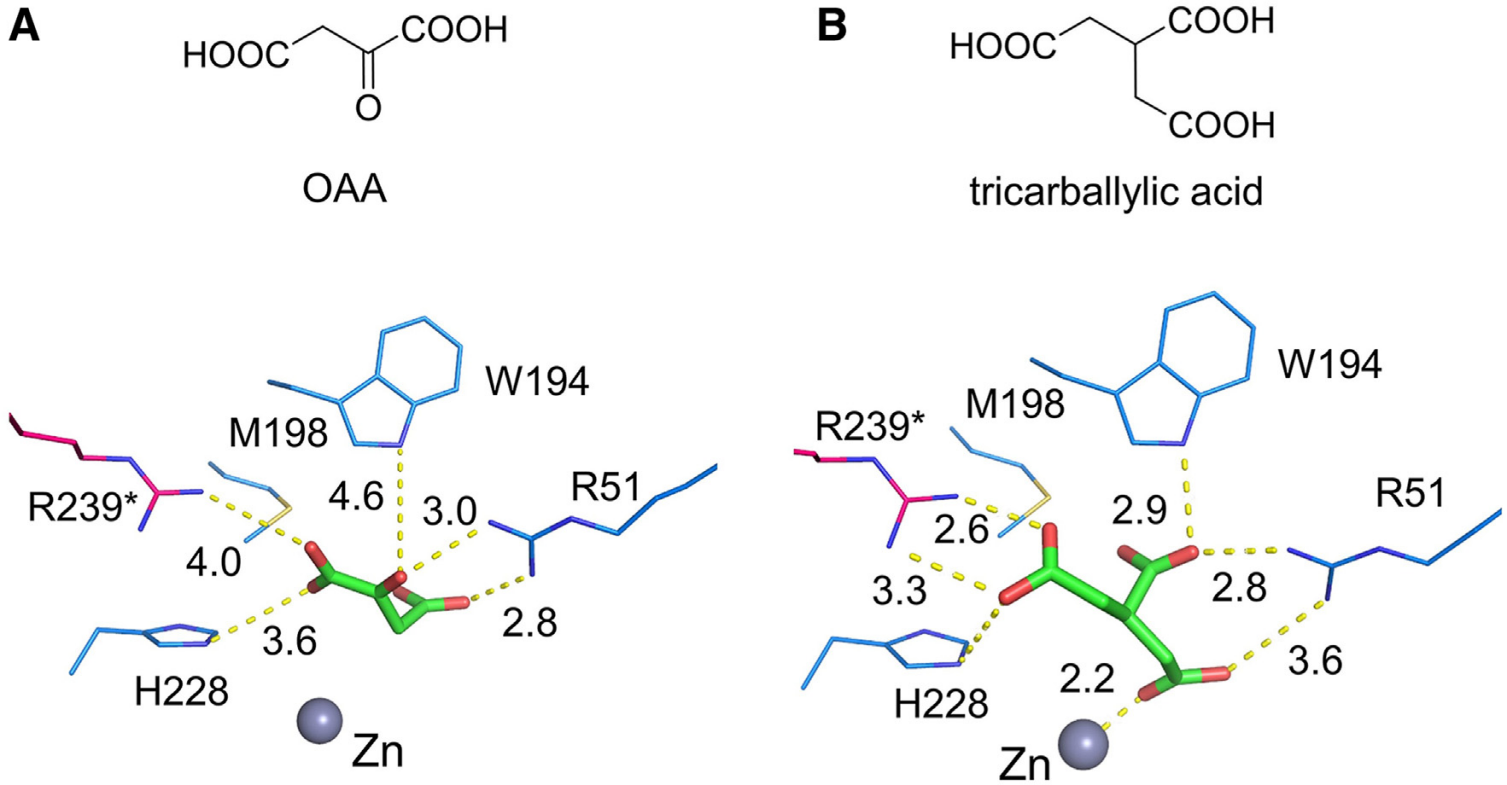
**The docking models of OAA and tricarballylic acid in ACMSD.** The chemical structures of OAA (*A*) and tricarballylic acid (*B*) are drawn on the top of the docking representations. The Interacting residues are shown as *blue* and *magenta sticks*. The Zn ions are shown in *gray balls*. The docked molecules are shown in *green sticks*. The distances are shown in *yellow dashes* and labeled in the unit of angstrom.

We docked OAA (Fig. 5*A*) and the extended carbon chain substrate analog, tricarballylic acid (Fig. 5*B*), into the active site. Both external ligands interact with the active site residues, Arg51, Arg239∗, Trp194, and His228, through their carboxylate groups, demonstrating that these are critical residues involved in binding and positioning substrate. Interestingly, the extra carboxylate group of tricarballylic acid extends into the active site and directly binds to the Zn ion, in contrast to ACMS-bound ACMSD, where the aldehyde group points out of the active site pocket. Replacement of the zinc-bound water with tricarboxylic acid likely inactivates ACMSD, as shown in the binding mode of the inhibitor, TES1025 (Fig. S1).

## Discussion

### Mechanistic insights from the malonate-bound structure

Given the rapid catalytic turnover of ACMSD and the instability of ACMS, co-crystallization of ACMS with the enzyme or its variants has proven challenging. To circumvent this limitation, we employed ACMS analogs that are resistant to decarboxylation by ACMSD.

The crystal structure of ACMSD complexed with malonate reveals that the two active site Arg residues, Arg51 and Arg239∗ from a neighboring subunit, form ion pairs with the two carboxylate groups of OAA. This observation aligns with previous mutagenesis and biochemical studies on R51A/K and R239A/K∗ mutants (28).

The malonate-bound structure provides valuable insights into the enzyme-substrate interaction and the catalytic mechanism. The formation of ion pairs between the Arg residues and the carboxylate groups of malonate highlights the importance of these residues in substrate recognition and binding. Additionally, the malonate-bound structure can serve as a model for understanding the potential binding mode of ACMS, despite the challenges associated with co-crystallization.

Like malonate, ACMS may also not directly coordinate with the metal ion in ACMSD. The malonate-bound structure demonstrates that the active site’s two catalytically essential Arg residues act as gatekeepers, preventing the substrate from ligating to the metal ion. This suggests that ACMS may similarly bind to the active site, with its C2=C3 double bond positioned towards the metal-bound water. As the zinc-bound water is likely deprotonated to form a reactive hydroxide ion, the positioning of ACMS in the active site is crucial for facilitating the decarboxylation reaction. The malonate-bound structure provides valuable insights into this mechanism by revealing the critical interactions between ACMS and the active site residues likely involved in substrate recognition and positioning for catalysis.

Inhibiting ACMSD decarboxylase activity has been shown to increase *de novo* NAD^+^ synthesis pathway (Fig. 1) and enhance mitochondrial function (14, 15, 16). This suggests that targeting ACMSD could be a promising therapeutic strategy for diseases or conditions associated with NAD^+^ deficiency or mitochondrial dysfunction. Several inhibitors of ACMSD have been identified, including 1,3-dihydroxyacetone phosphate (DHAP), PDC, and TES-1025. These inhibitors bind to the ACMSD active site and compete with the substrate for binding. PDC and DHAP share a similar binding mode to malonate, where their carboxylate groups interact Trp191, Arg47, and Arg235∗ in human ACMSD (23), corresponding to Trp194, Arg51, and Arg239∗, respectively (Fig. S1). TES-1025 binds directly to the Zn ion (31), raising the concern that it might bind the zinc center of other enzymes.

Understanding the binding modes of these inhibitors can provide valuable insights for developing novel ACMSD inhibitors with improved potency and selectivity. Additionally, identifying new inhibitors could help to expand the therapeutic potential of targeting ACMSD.

### ACMSD-catalyzed OAA tautomerization

OAA is unique in its ability to bind to the ACMSD active site without undergoing decarboxylation. While not a natural substrate, OAA serves as a valuable tool for studying ACMSD’s catalytic mechanism. The shorter chemical structure of OAA, lacking the aldehyde end, demonstrates the flexibility of ACMSD’s active site and its ability to accommodate different substrate analogs. This information contributes to a broader understanding of ACMSD’s substrate specificity and catalytic mechanism. Interestingly, OAA undergoes an unexpected keto-enol tautomerization in the presence of ACMSD (Fig. 2). This finding gains further insights into the enzyme’s catalytic versatility and potential interactions with other substrates or cofactors.

**Figure 6:**
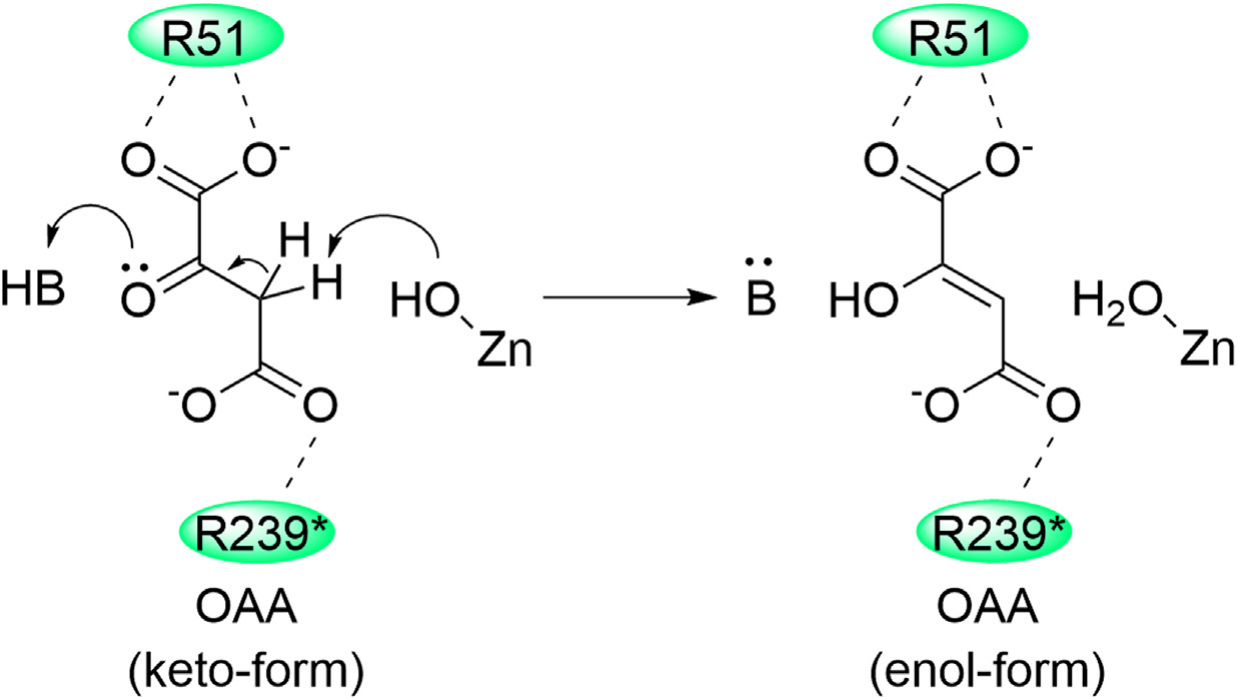
**Proposed mechanism of OAA tautomerization catalyzed by ACMSD**.

Mutating Arg51 or Arg239 to alanine abolished the tautomerization activity on OAA, highlighting the essential role of both arginine residues in catalysis. We propose a mechanism (Fig. 6) wherein ACMSD binds OAA, forming salt bridges with its two carboxylic groups through Arg51 and Arg239 during tautomerization. Subsequently, a nearby base deprotonates the C3 carbon, while an acid protonates the carbonyl oxygen of the keto form of OAA, resulting in the production of its enol form as the product. Despite the structural proximity of Trp194 and His228 to the active site, they are unlikely to be the base, as demonstrated by the comparable tautomerization activity of H228A and W194A mutants compared to the wild-type ACMSD (Table 1).

H228Y completely eliminates the tautomerization activity, possibly due to the bulkier side chain of tyrosine affecting OAA binding in the active site. Interestingly, previous reports indicate that H228Y prefers to bind to iron ions in the active site rather than zinc ions (30). Due to the coordination preference, the metal-bound water is absent in the Fe-bound ACMSD H228Y structure (PDB ID: 4ERG). This suggests that water molecules bound to metals may be critical for the tautomerization process. Notably, the essential residues found in ACMSD across both eukaryotes and prokaryotes indicate a high degree of conservation of these residues (Fig. S4).

While both kinetic analyses and crystal structures offer valuable insights into the catalytic roles of His228, Trp194, Arg51, and Arg239, changes in stability and dynamics may affect the interpretation of the activity data measured on the mutants. This understanding is crucial for elucidating both the catalytic and dynamic properties of ACMSD. Additional protein dynamics simulations are necessary to determine whether mutations in these residues affect protein dynamics or the conformational ensemble in future studies.

While the unexpected catalytic activity on OAA deepens our understanding of ACMSD’s catalytic versatility, the biological relevance of OAA tautomerization remains to be fully elucidated. OAA in cells is consumed primarily by citrate synthase in the TCA cycle and phosphoenolpyruvate carboxykinase (PCK) gluconeogenesis metabolic pathway.

In its keto form, OAA, is a central intermediate in the tricarboxylic acid cycle (TCA) for citrate synthesis under the catalysis of citrate synthase. However, ACMSD’s isomerase activity on OAA could potentially influence the distribution of OAA between the TCA cycle and gluconeogenesis. Further investigations are needed to determine the physiological significance of ACMSD’s isomerase activity on OAA and its potential impact on cellular metabolism.

The *K*_M_ value of OAA in the citrate synthase reaction is under 10 μM, which maintains OAA’s concentration under a micromole level (34). In contrast, ACMSD catalyzes OAA tautomerization with a *K*_M_ value of 1120 ± 140 μM, making it less competitive to regulate the keto and enol form ratio of OAA. Notably, citrate synthase operates in the mitochondria; human ACMSD does not have the mitochondrial targeting sequence and most likely functions in the cytosol. These facts suggest a limited connection between the tautomerization activity of ACMSD and the TCA cycle.

While the direct connection between ACMSD’s tautomerization activity and the TCA cycle may be limited, OAA is a key metabolic intermediate present in both the mitochondria and the cytosol. Its levels can be influenced by various conditions or disease states, leading to elevated cytosolic OAA (35, 36, 37). For example, mitochondrial dysfunction can disrupt the TCA cycle, resulting in increased OAA accumulation in the cytosol. Additionally, conditions such as PCK deficiency in cancer cells (38) and certain liver diseases can lead to elevated OAA levels due to impaired OAA metabolism (39, 40, 41). The enol form of OAA is a metabolically inactive form that is a strong inhibitor of succinate dehydrogenase (42). Therefore, the potential isomerase activity of ACMSD on OAA warrants further investigation in future biological studies. By understanding the role of ACMSD in OAA metabolism, we may gain insights into potential therapeutic strategies for diseases or conditions associated with altered OAA levels.

### Comparison of ACMSD with the tautomerase superfamily

Upon aligning protein sequences and superimposing three-dimensional structures, ACMSD appears distinct from most of the tautomerase superfamily. Nevertheless, an intriguing similarity emerges between them. In the tautomerase superfamily, exemplified by enzymes like 4-oxalocrotonate tautomerase and OAA tautomerase (43), arginine residues within the active site serve to anchor the one or two carboxylate groups of substrates, facilitating subsequent catalytic reaction with assistance from nearby residues (44). This binding mode is reminiscent of what was observed in the crystal structures of ACMSD in complex with malonate and pyridine-2,6-dicarboxylic acid (Figs. 3 and S1) (23). Here, we simulated the binding mode of ACMS in the active site, where Arg51 and Arg239∗ interact with the two carboxylate groups of the substrate (Fig. 4*B*). Notably, the aldehyde group on ACMS is positioned opposite the Zn center, as it is not directly involved in decarboxylation.

Furthermore, tautomerases usually exhibit high-order oligomers in their quaternary structures, with essential arginine residues located at the adjacent units (45, 46). In essence, the multimeric form is important for catalyzing tautomerization reactions. As previously mentioned, Arg239∗ from the adjacent protomer is critical in decarboxylation. Therefore, ACMSD likewise displays concentration-dependent catalytic activity, whereby a higher enzyme concentration yields high-order oligomers and, consequently, higher activity (28, 29). Given that ACMSD shares key features with tautomerases, this lays a structural foundation for explaining the finding that ACMSD exhibits a tautomerization activity. Furthermore, it is conceivable that enzymes similar to ACMSD within the amidohydrolase superfamily might also possess tautomerization activity on specific substrates.

### A proposed mechanism involving ACMS tautomerization

In the tryptophan-kynurenine pathway, three enzymatic reactions involve unstable substrate, product, or both. The step before ACMSD is a non-heme iron-mediated dioxygenase, 3-hydroxyanthranilic acid dioxygenase (HAO), that opens the phenolic ring of the substrate 3-hydroxyanthranilic acid to generate the unstable product ACMS, which has 32 possible (16 enol and 16 keto) tautomers (8). A hidden enzymatic isomerization converts its immediate dioxygenase product, 3*E*,5*Z*,2*t*,4*c*-enol tautomer of ACMS, to 3*E*,5*Z*,2*t*,4*t*-enol tautomer before releasing it from the iron center, as demonstrated by a series of catalytic intermediate structures of the reaction. The Fe center and Arg99 in the active site of HAO anchor the two carboxylic groups of ACMS. It is plausible to speculate that ACMSD may exhibit tautomerization activity on one of the ACMS tautomers. The enzymatic reaction downstream of ACMSD involves a dehydrogenase that oxidizes the aldehyde group of 2-AMS to the carboxylic group. Previous studies have captured an imine/enol tautomer intermediate *in crystallo*, indicating that substrate isomerization precedes dehydrogenation (7, 9). Within the active site, the dehydrogenase, like ACMSD shown in this study, also employs two arginine residues to bind the dicarboxylic group of 2-AMS. Therefore, the enzymes in the tryptophan-kynurenine pathway typically feature one or two arginine residues that interact with the dicarboxylic group of the substrate, facilitating the proper positioning of the substrate through isomerization and preventing undesired auto-cyclization in the active site to enable the desired reaction.

**Figure 7:**
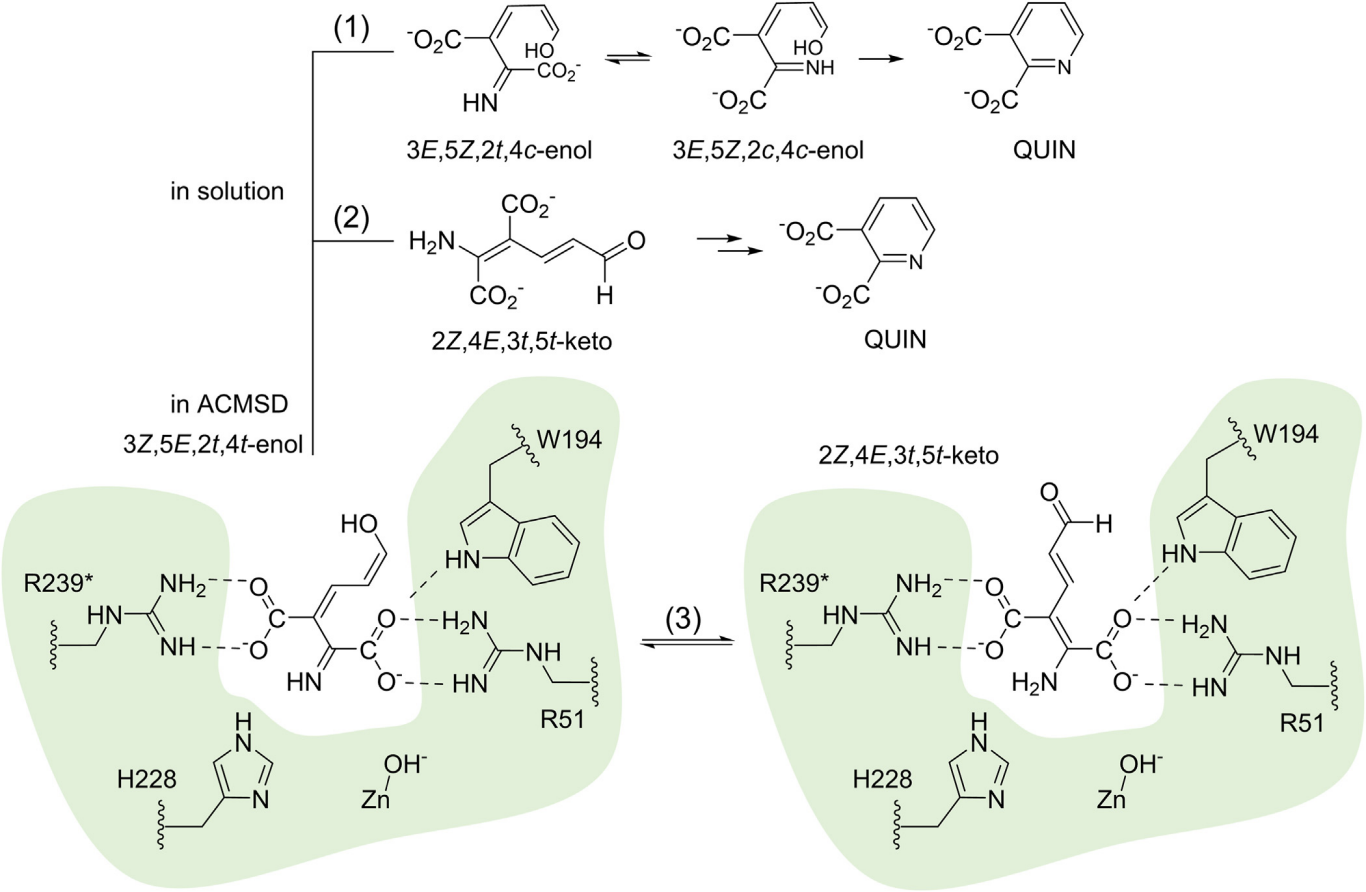
**The enol-form ACMS will either be self-tautomerized to QUIN in solution (1 and 2) or be ACMSD-mediated tautomerized to the keto form precedes decarboxylation (3) after leaving the active site of HAO**.

Inspired by the mechanistic studies of the immediate neighboring enzymes in the tryptophan-kynurenine pathway, we propose that ACMSD-catalyzed decarboxylation can involve the tautomerization of ACMS, should an undesired tautomer arrive at the active site of ACMSD. As mentioned above, the product of HAO has previously been identified as 3*E*,5*Z*,2*t*,4*t*-enol form by crystallography (8). After leaving the active site of HAO, the enol form will be auto-tautomerized to 3*E*,5*Z*,2*c*,4*c*-enol form and cyclized to QUIN (Fig. 7), as the enol form is the preferred to form QUIN compared to the keto form (13). On the other hand, the enol-form ACMS possibly converts to 2*Z*,4*E*,3*t*,5*t*-keto form, the only tautomer form detected in solution by NMR (13), and cyclizes to QUIN eventually. However, the plausible scenario is that, instead of staying in solution, the enol form directly goes into the active site of ACMSD and enzymatically converts to a keto-form with assistance by Arg51, Arg239∗, Trp194, and H228 before the decarboxylation of ACMS occurs (Fig. 7), which may prevent the auto-cyclization of ACMS to QUIN. The presumable tunnel linking the active sites of HAO and ACMSD has recently been proposed to regulate the metabolites precisely (6). Subsequently, the metal-bound hydroxide ion conducts a nucleophilic attack on the C2=C3 double bond of ACMS, initiating the decarboxylation reaction (22).

In summary, we elucidated the substrate-binding network in ACMSD through structural and kinetic assays. Moreover, we found a previously unknown isomerase activity of ACMSD that can catalyze the enol-keto tautomerization of oxaloacetic acid, a common metabolite in all living species. However, ACMSD’s isomerase activity on OAA is unlikely to intertwine with OAA metabolism and the TCA cycle. The observation of the tautomerization of OAA and the similarities of ACMSD with 4-oxalocrotonate tautomerase and dedicated oxaloacetic acid tautomerase suggest that ACMSD can facilitate ACMS tautomerizations among distinct isoforms. This study deepens the understanding of ACMS-ACMSD interactions and the regulation of QUIN in the tryptophan-kynurenine pathway in depth. Thus, the findings will inform the therapeutic design of ACMSD inhibitors.

## Experimental procedures

### Materials

The chemicals, including 3-hydroxyanthranilic acid, oxaloacetic acid, malonic acid, succinic acid, malic acid, and ammonium tartrate were purchased from Sigma-Aldrich. Tacsimate (pH 7.0) was purchased from Hampton Research.

### Protein preparation

The heterogeneous expression and purification strategy of ACMSD from *P. fluorescens* KU-7 (UniProt ID: Q83V25) were described in the previous publication (28). The purified ACMSD was stored in a desalting buffer containing 25 mM HEPES pH 7.0 for the activity assay and crystallization.

### Site-directed mutagenesis

The mutants R51A, R239A, and H228Y were obtained from previous publications (28, 30). The mutants were constructed using the PCR overlap extension mutagenesis method. The recombinant plasmid pET16b-ACMSD was used as the template, and the forward primer are 5′-GAAAAAGTGGATGTTGCCGGCGCTGGTGGCGATGCCAGCAG-3′ for W194A and 5′-CAAGATCTGTTTCGGGGCGGGTGGGGGAAGTTTCG-3′ for H228A. The procedures of expression and purification of mutants were the same as the wild-type ACMSD.

### Activity assay of ACMSD towards ACMS and analogs

NMR was used to analyze the possible decarboxylation activity of ACMSD on the substrate analogs itaconic acid and mesaconic acid. The analog itaconic acid or mesaconic acid was solved in a water solution containing 10% D_2_O. The NMR spectra were recorded on a Bruker (Billerica, MA) Avance III HD 500 MHz spectrometer equipped with a 5 mm Cryoprobe Prodigy apparatus at 300 K. One-dimensional ^1^H spectra were recorded with eight scans. The NMR data were processed by the software of MestReNova NMR v11.0.3. The reaction mixture containing 11 mM itaconic acid or mesaconic acid was mixed with 20 μM ACMSD for 12 h, measured by NMR, and shown in Fig. S2.

The substrate ACMS was prepared with an enzymatic method described previously (25, 47). The activity of ACMS decarboxylation was measured by tracking the decrease in absorbance at 360 nm (ε_360_ = 47,500 cm^−1^M^−1^) at room temperature with an Agilent 8453 diode-array spectrophotometer. Similarly, the activity of OAA tautomerization was measured by monitoring the increase in absorbance at 260 nm at the HEPES buffer pH 7.0 (32, 33). The reaction rates (*V*_*o*_) *versus* gradient substrate concentrations [*S*] were fitted with the Michaelis-Menten equation and generated the kinetic parameters, *k*_cat_ and *K*_M_. All samples were analyzed in triplicate independently.

(1) $$V_{o}/\left[E\right]=\frac{k_{\text{cat}}\times \left[S\right]}{K_{M}+\left[S\right]}$$

### Crystallization and structure determination of wild-type ACMSD and W194A

The concentration of ACMSD and W194A for crystallization was 10 mg/ml (270 μM). The crystallization of wild-type ACMSD was first attempted by using the Crystal Screen Kits. The initial condition contains 2% (*v/v*) Tacsimate pH 7.0, 0.1 M sodium citrate tribasic pH 5.6, and 12% (*w/v*) polyethylene glycol 3350. Then, Tacsimate was separately replaced with each component for further refinement, including malonic acid, succinic acid, malic acid, and tartrate. As a result, replacing it with malonate (50 mM) yielded diffractable crystals.

The X-ray diffraction datasets of ACMSD were collected, processed, and scaled by HKL2000 (48) at the Stanford Synchrotron Radiation Lightsource (SSRL) and Structural Biology Center (SBC). The structures were solved using molecular replacement with the wild-type ACMSD (PDB ID: 2HBV) as the template and further processed using the graphical interface of *phenix.refine* within the Phenix 1.17.1 (49) and COOT 0.8.3 (50). The figures were drawn with PyMOL (51). The detailed information is summarized in Table 2. Each unit cell contains eight asymmetric units in ACMSD (Fig. S5). ACMSD has been identified as the dimeric form (28). The interacting residues located at the monomer-monomer interface in the dimeric ACMSD were highlighted in Fig. S6.

### Structural modeling of ACMSD

The substrate, ACMS, was docked into the active site of ACMSD using the *Discover Studio* 2020 (DS2020). The malonate-bound ACMSD (PDB ID: 8YT1) was applied as the reference. The binding site is a globular shape with a radius of 10 Å whose origin was set to be (−48.3, 4.2, −14.7). The flexible molecular docking was performed in the CDOCKER Module in DS2020 in which the conformation method was the BEST. Results were picked up based on the docking scores and the binding mode is visualized by PyMOL program.

## Data availability

The crystal structures have been deposited in the RCSB Protein Data Bank (PDB) under accession codes 8YT1 and 8YT2. The article and the supporting information contain all other data.

## Supporting information

This article contains supporting information.

## Conflict of interest

The authors declare that they have no conflicts of interest with the contents of this article.
