# Supplementary material for: α-Amino-β-carboxymuconate-ε-semialdehyde decarboxylase catalyzes enol/keto tautomerization of oxaloacetate
Source: J Biol Chem. 2024 Oct 11;300(11):107878. doi: 10.1016/j.jbc.2024.107878 (PMC11650718; doi:10.1016/j.jbc.2024.107878)
Supplement: Supplementary Information [file mmc1.docx]

**Supporting Information**

**α-Amino-β-Carboxymuconate-ε-Semialdehyde Decarboxylase Catalyzes Enol/Keto Tautomerization of Oxaloacetate**

Yu Yang^1,2,*^, Ian Davis^2^, Ryan A. Altman^3^, and Aimin Liu^2,*^

^1^State Key Laboratory of Biocatalysis and Enzyme Engineering, Hubei Collaborative Innovation Center for Green Transformation of Bio-Resources, Hubei Key Laboratory of Industrial Biotechnology, School of Life Sciences, Hubei University, Wuhan, China

^2^Department of Chemistry, University of Texas at San Antonio, San Antonio, Texas, United States

^3^Borch Department of Medicinal Chemistry and Molecular Pharmacology and Department of Chemistry, Purdue University, West Lafayette, Indiana, United States

*****Corresponding authors:

E-mail: yangyu@hubu.edu.cn or Feradical@utsa.edu

Present address for Ian Davis: Diagnostic Systems Division, United States Army Medical Research Institute for Infectious Diseases, Fort Detrick, Maryland, United States

Table of Content

**Figure S1**. Comparison of the ligand-bound crystal structures of ACMSD

**Figure S2**. Detection of possible decarboxylation activity of ACMSD on itaconic acid, mesaconic acid, and OAA

**Figure S3**. Structural superposition of ACMSD crystal structures

**Figure S4**. The sequence alignment of ACMSD from eukaryotes and prokaryotes.

**Figure S5**. A unit cell of the crystal structure of ACMSD

**Figure S6**. The essential interacting residues at the monomer-monomer interface

**Table S1**. Detection of possible decarboxylation activity of ACMSD on OAA

**Table S2**. Structural alignment parameters

**Table S3**. Distances between ligand (ACMS/malonate) and key residues in ACMSD

**Figure S1**. **Comparison of the ligand-bound crystal structures of ACMSD**. The PDC-bound (PDB ID: 4IH3), DHAP-bound (PDB ID: 2WM1), diflunisal-bound (PDB ID: 7K12), and TES-1025-bound (PDB ID: 7PWY) ACMSD structures are colored in cyan, green, magenta, and yellow, respectively. The residue with a superscript is an intruding residue from the neighboring subunit. The distances between atoms are labeled in the unit of Angstrom.


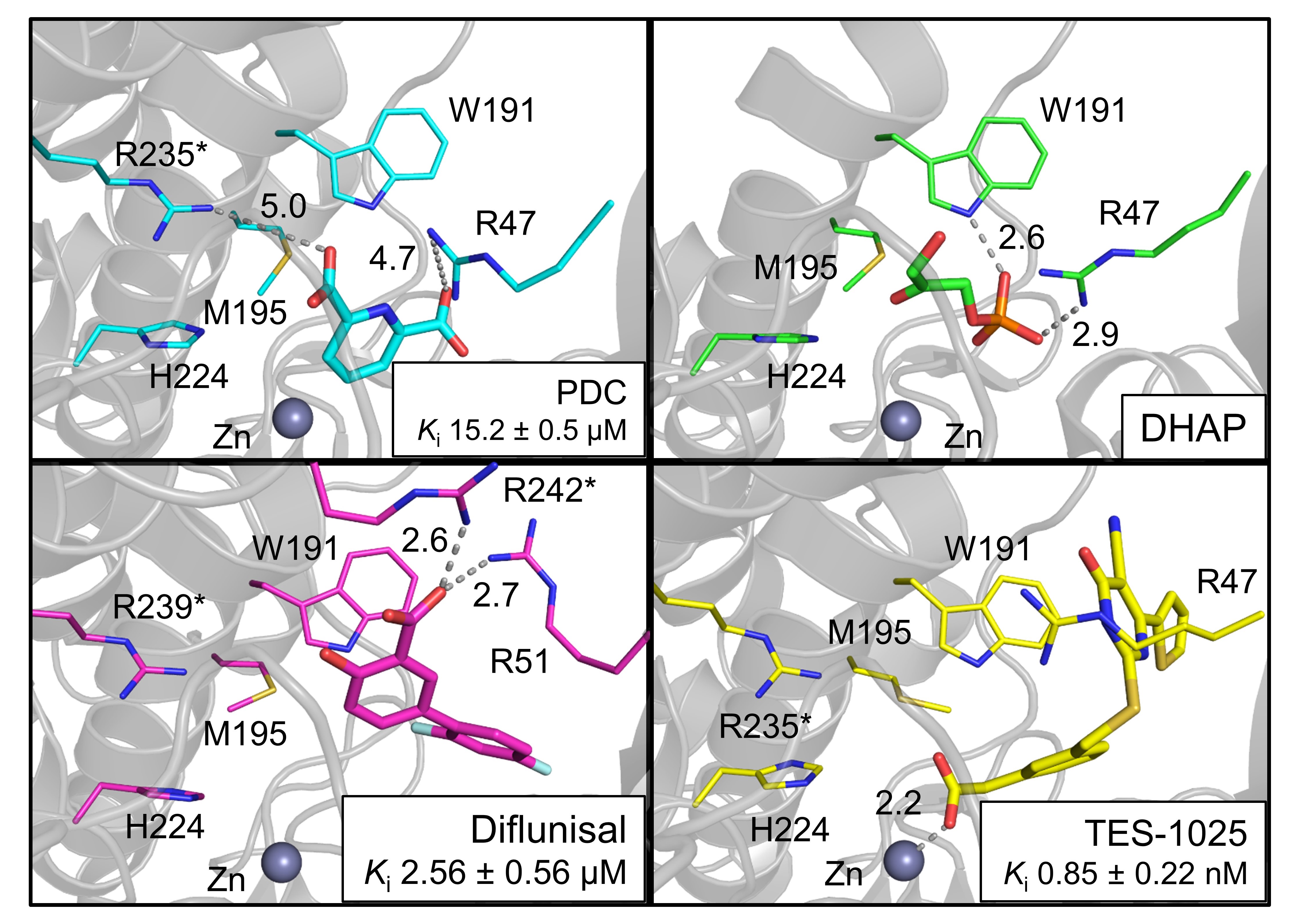


**Figure S2. Detection of possible decarboxylation activity of ACMSD on itaconic acid, mesaconic acid, and OAA. A)** NMR assay of ACMSD on itaconic acid and mesaconic acid. The analog itaconic acid or mesaconic acid was solved in the water solution (10% D_2_O). The reaction mixture containing 11 mM itaconic acid or mesaconic acid was mixed with 20 μM ACMSD for 12 hours and measured by NMR. The protons on analogs were labeled on the NMR profiles, showing no observable differences after mixing with the enzyme ACMSD. **B**) Detection of possible decarboxylation activity of ACMSD on OAA. The assay was performed by mixing excessive ACMSD (10 μM) and OAA (0.7 mM) at room temperature. The decomposition rate of OAA was measured by recording a decrease in the absorbance at 260 nm for 10 hours. As a result, the decomposition rate of OAA was not significantly improved by adding ACMSD, indicating that ACMSD cannot mediate the decarboxylation of OAA.


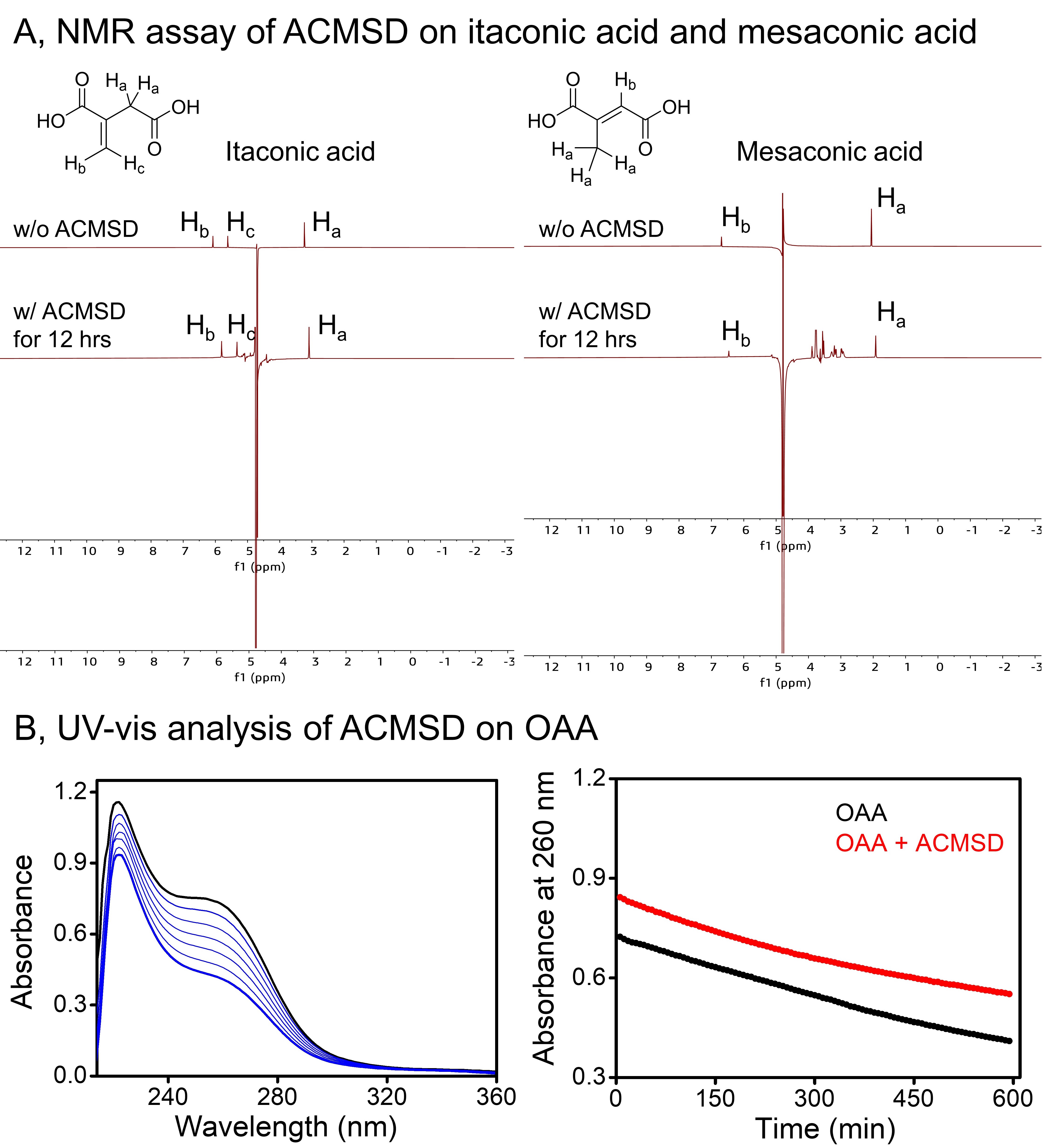


**Figure S3**. **Superposition of ACMSD structures**. A) The holo ACMSD (gray, PDB ID: 2HBV) with malonate-bound ACMSD (blue, PDB ID: 8YT1) structures, and **B**) holo ACMSD with mutant W194A ACMSD (pink, PDB ID: 8YT2) structures.


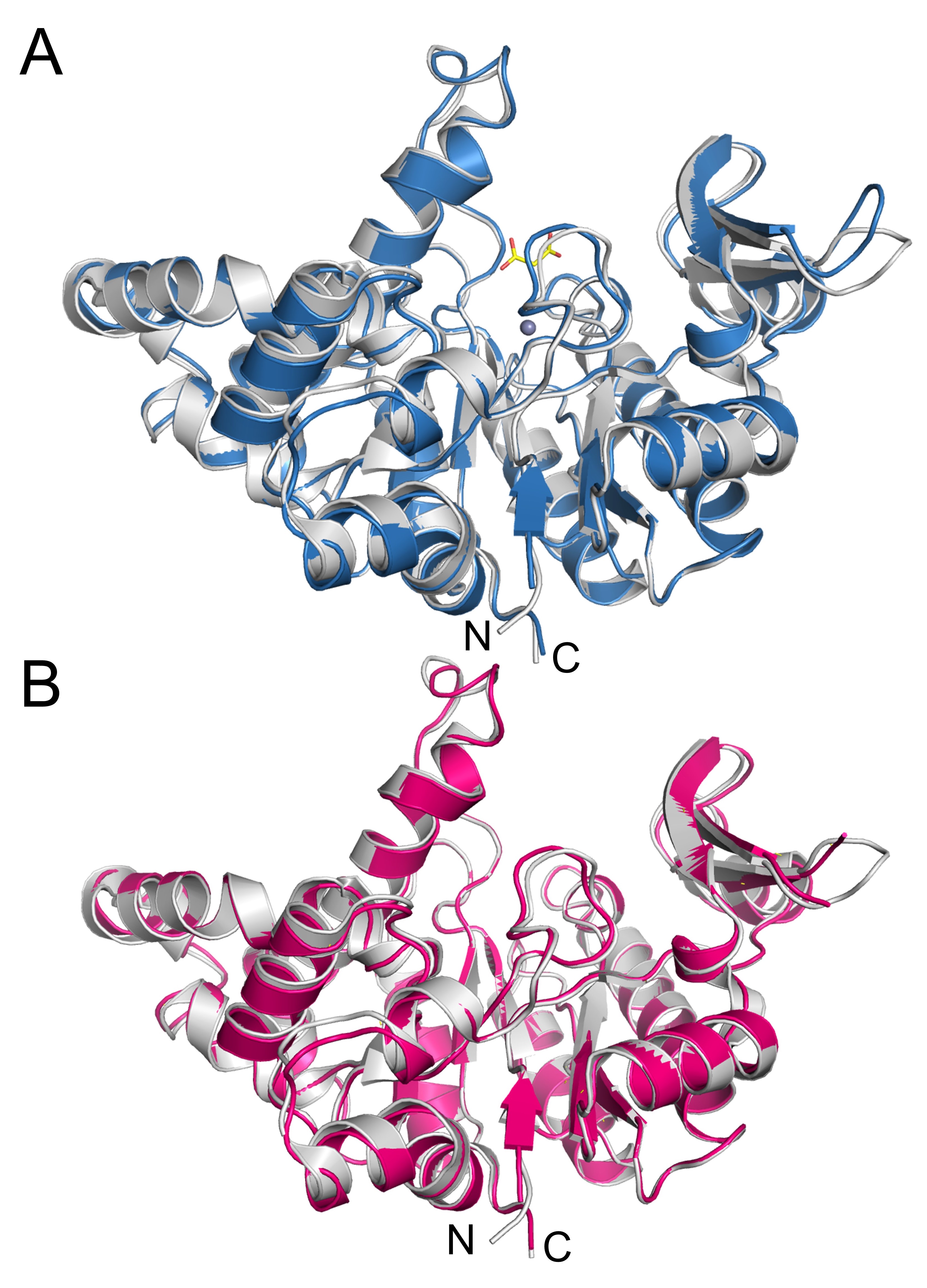


**Figure S4. The sequence alignment of ACMSD from eukaryotes and prokaryotes.** The essential residues are marked by green stars. The sequence IDs of ACMSD are NP_001028213 for *Mus musculus*, XP_008256730 for *Oryctolagus cuniculus*, XP_002671211 for *Naegleria gruberi*, XP_004987954 for *Salpingoeca rosetta*, XP_022325457 for *Crassostrea virginica*, XP_025871357 for *Vulpes vulpes*, XP_025171184 for *Rhizophagus irregularis*, XP_022425302 for *Delphinapterus leucas*, XP_005868918 for *Myotis brandtii*, WP_017134319 for *Pseudomonas agarici*, WP_046803787 for *Achromobacter* sp., WP_069221255 for *Burkholderia multivorans*, WP_130360259 for *Pigmentiphaga kullae*, WP_028222400 for *Paraburkholderia oxyphila*, WP_130008903 for *Pusillimonas ginsengisoli*.


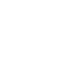
**
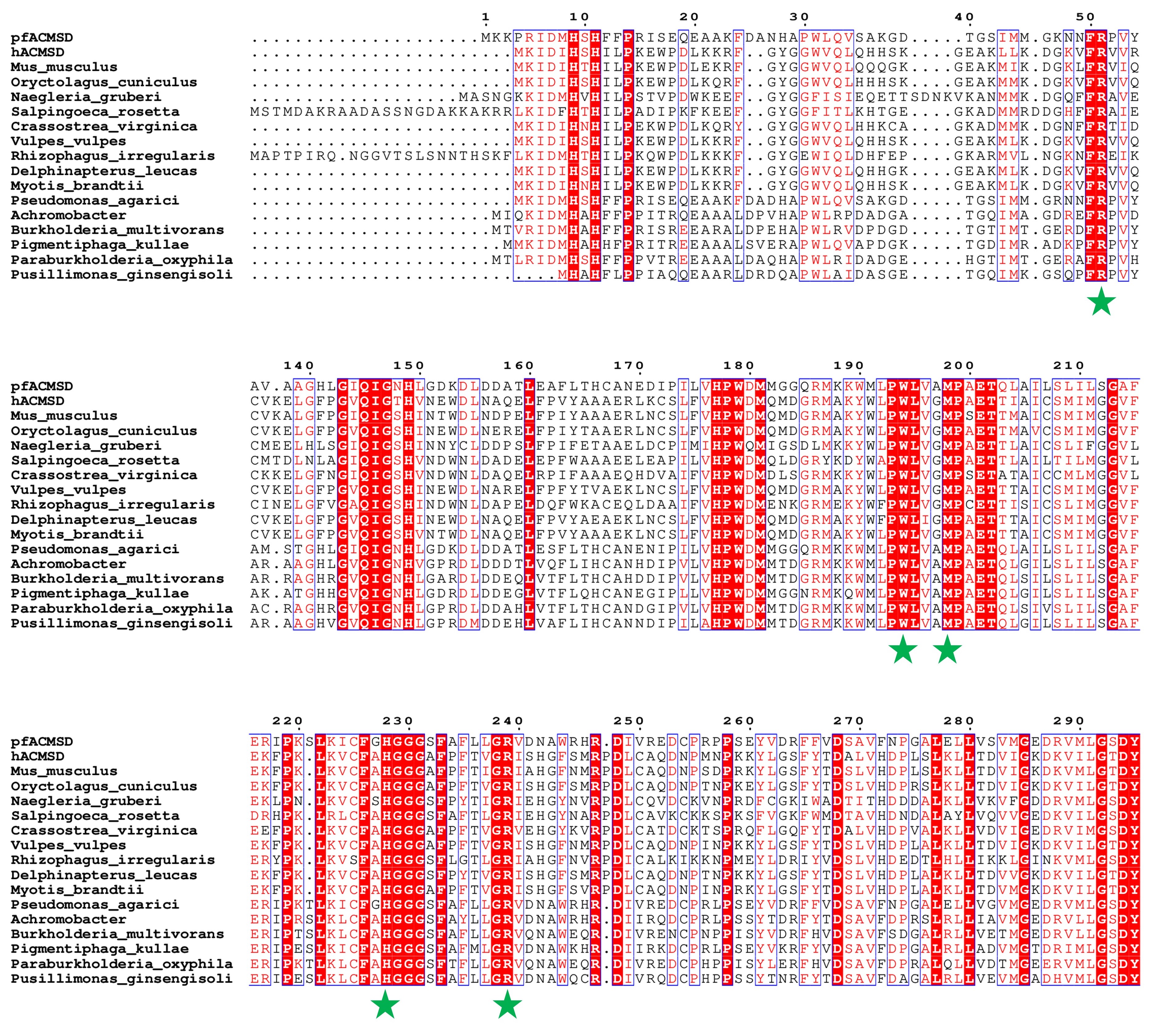
**

**Figure S5. A unit cell of the crystal structure of ACMSD**. Each unit cell contains 8 asymmetric units.


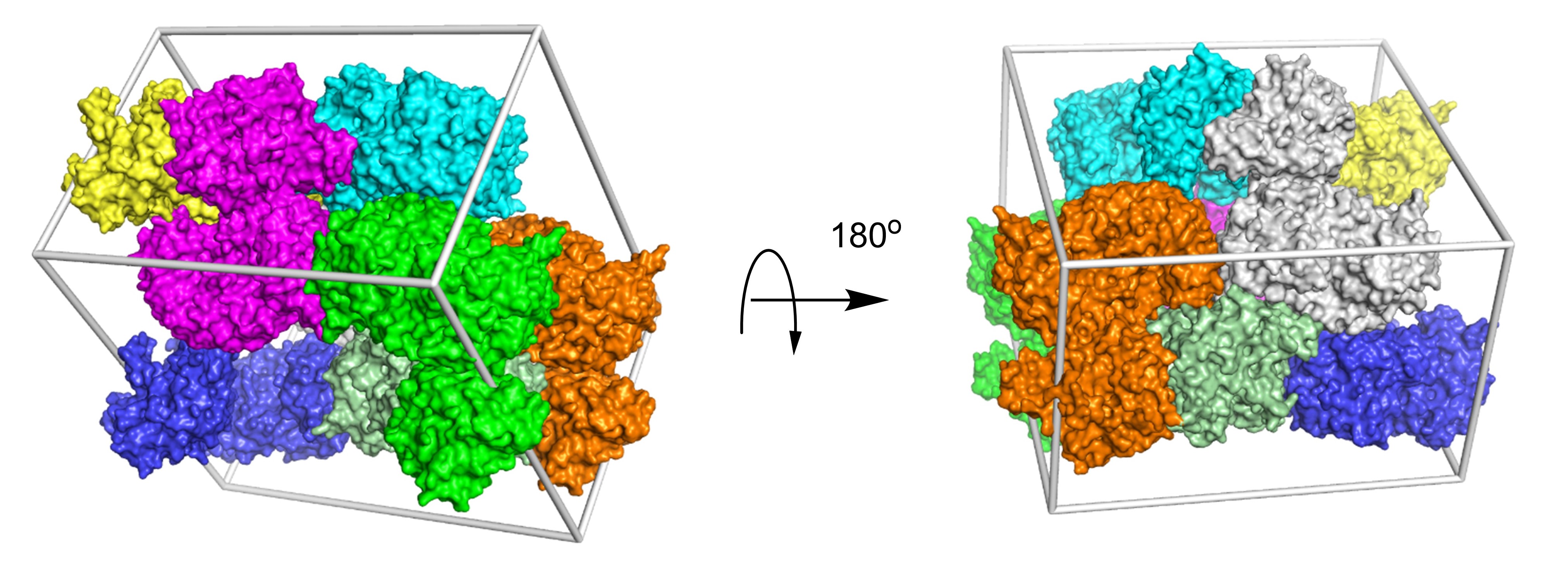


**Figure S6. The essential interacting residues at the monomer-monomer interface**. The two monomers are labeled as magenta and light blue, respectively. The interacting residues are shown in the sticks and labeled. The yellow dash lines show the distances within 4 Å. The black arrows point to the active site of ACMSD. The inter-subunit residue distances are summarized on the bottom.


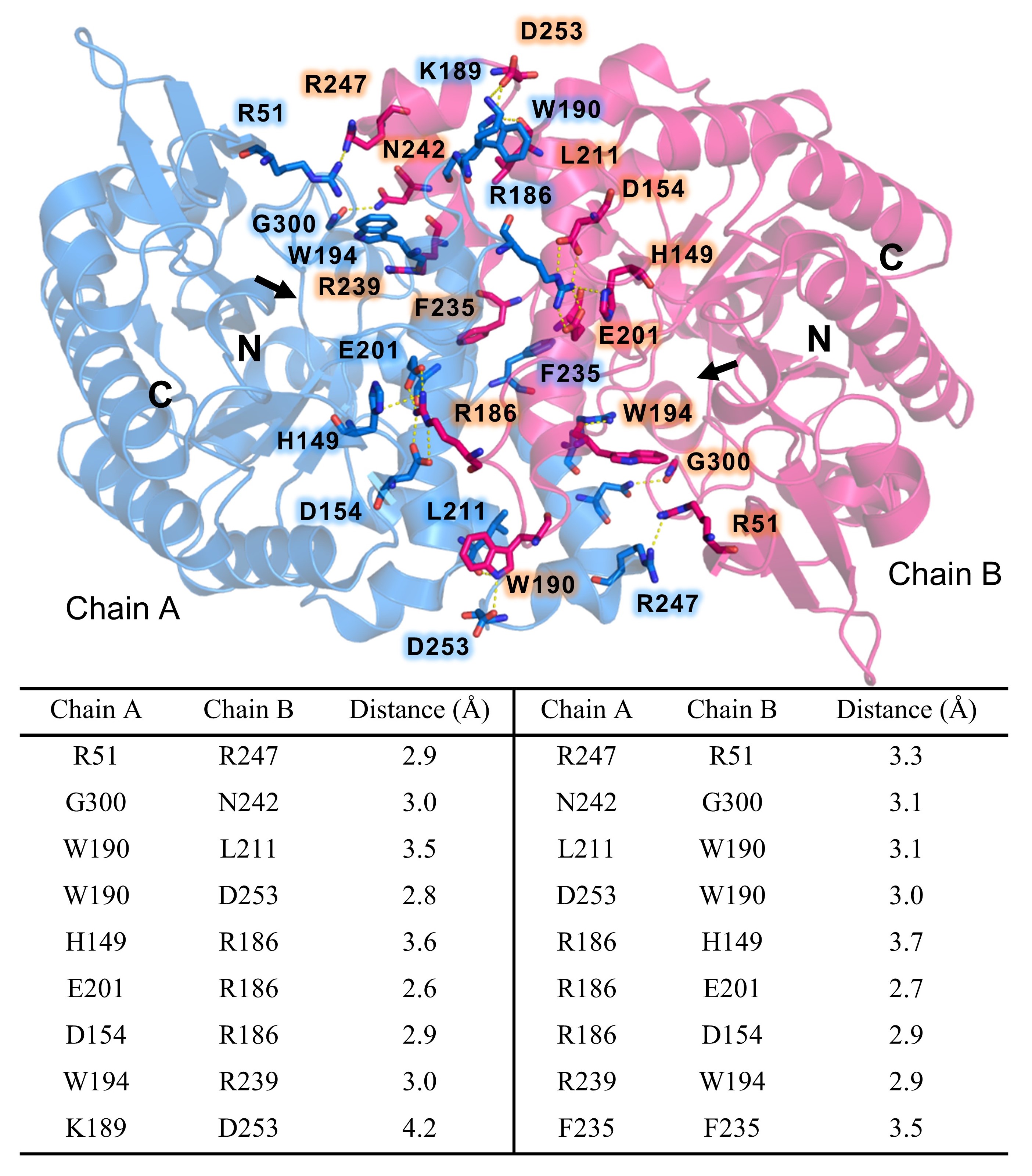


**Table S1**. **Detection of possible decarboxylation activity of ACMSD on OAA.**

|  | **-AU/s * 10^6^** | |
| --- | --- | --- |
|  | OAA | OAA, w/ 10 μM ACMSD |
| 265 nm | 9.03 ± 0.02 | 9.74 ± 0.10 |
| 290 nm | 2.07 ± 0.01 | 2.50 ± 0.03 |

**Table S2**. **Structural alignment parameters**. The structures used for superimposition are in **Figure S3**. The PDB IDs are in parentheses.

| **Target structure** | **Reference structure** | **RMSD values/Å** | **Numbers**  **of Cα** |
| --- | --- | --- | --- |
| Malonate-bound ACMSD (8YT1) | Holo ACMSD (2HBV) | 0.276 | 258 |
| ACMSD W194A (8YT2) | Holo ACMSD (2HBV) | 0.265 | 265 |

**Table S3**. **Distances between ligand (ACMS/malonate) and key residues in ACMSD**

| Residues | ACMS | | Malonate | | Reference |
| --- | --- | --- | --- | --- | --- |
|  | Modeling | | PDB ID: 8YT1 | |  |
|  |  | |  | |  |
| Arg51 | NH-O_C7_ | 2.8 Å | NH-O_C3_ | 2.4 Å | *J. Biol. Chem.* 288, 30862-30871 |
|  | NH_2_-O_C7_ | 2.9 Å | NH_2_-O_C3_ | 3.3 Å |  |
| Arg239* | NH-O_C1_ | 3.0 Å | NH-O_C1_ | 3.1 Å |  |
|  | NH_2_-O_C1_ | 2.8 Å | NH_2_-O_C1_ | 3.8 Å |  |
| His228 | N_E2_- O_C3_ | 3.6 Å | N_E2_- O_C1_ | 3.0 Å | *Biochemistry* 2012 51, 5811-5821 |
| Trp194 | N_E1_- O_C7_ | 2.9 Å | N_E1_- O_C3_ | 3.4 Å | In this study |

*Arg239 is an intruding residue from the neighboring subunit (chain B), while other residues listed are from chain A.
